# Assessment of the RNA*Sound* RNA Sampling Card for the Preservation of Influenza Virus RNA

**DOI:** 10.3389/fmicb.2016.01736

**Published:** 2016-11-02

**Authors:** Hilda Lau, Aeron C. Hurt

**Affiliations:** ^1^WHO Collaborating Centre for Reference and Research on Influenza, Victorian Infectious Diseases Reference Laboratory, Peter Doherty Institute for Infection and Immunity, MelbourneVIC, Australia; ^2^Melbourne School of Population and Global Health, The University of Melbourne, MelbourneVIC, Australia

**Keywords:** RNA*Sound* card, influenza virus, RNA preservation, room temperature, real time RT-PCR, Sanger sequencing

## Abstract

Shipping influenza virus specimens, isolates or purified RNA is normally conducted at ultra-low temperatures using dry ice to ensure minimal degradation of the samples but this is expensive and requires special packaging and shipping conditions. Therefore, alternative methods for shipping influenza viruses or RNA at ambient temperatures would be desirable. The RNA*Sound* RNA Sampling Card (FortiusBio LLC, San Diego, CA, USA) is a device that enables specimens or isolates to be applied to a card, whereby viruses are inactivated, while RNA is preserved and purified RNA can also easily be eluted. To evaluate this card, we applied influenza virus cell culture isolate supernatants to either the RNA*Sound* card or Whatman Grade No. 1 filter paper (GE Healthcare, Rydalmere, NSW, Australia) and compared the preservation to that of material stored in liquid form. Preservation was tested using influenza A and B viruses at two different storage temperatures [cool (2–8°C) or room temperature (18–22°C)] and these were compared with control material stored at -80°C, for 7, 14, or 28 days. The quality of the RNA recovered was assessed using real time RT-PCR and Sanger sequencing. The RNA*Sound* card was effective in preserving influenza RNA at room temperature for up to 28 days, with only a minor change in real-time RT-PCR cycle threshold values for selected gene targets when comparing between viruses applied to the card or stored at -80°C. Similar results were obtained with filter paper, whilst virus in liquid form performed the worst. Nevertheless, as the RNA*Sound* card also has the capability to inactivate viruses in addition to preserving RNA at room temperature for many weeks, this makes it feasible to send samples to laboratories using regular mail, and thus avoid the need for expensive shipping conditions requiring biohazard containers and dry ice. Moreover, the quick and simple RNA recovery from the RNA*Sound* card allows recipient labs to obtain RNA without the need for special reagents or equipment.

## Introduction

Viruses that infect humans and animals can have a devastating impact on morbidity and mortality and therefore rapid analysis of specimens to determine the identity of causative viruses is important. Molecular-based assays, such as real-time RT-PCR, are rapid, sensitive and specific and are now widely used in diagnostic laboratories around the world (Espy et al., 2006). However, such specialized laboratories may be a large distance from the point of specimen collection, and as a result, shipment of samples can take several days. RNA viruses can be chemically unstable and susceptible to ubiquitous RNases in the environment that can degrade the sample, potentially affecting the ability of a laboratory to effectively analyze a sample and make a diagnosis (Buckingham and Flaws, 2007). Therefore, it is recommended that virus samples are stored in virus transport media and kept cold, or ideally, frozen at -80°C for shipment. However, this requirement has meant that shipment of samples from the field or remote hospital sites typically requires the use of dry ice which, because it is considered a ‘dangerous good’ by the International Air Transport Association (IATA), requires special packaging and shipping conditions resulting in high shipping costs (NPAAC, 2013). There is therefore a need for methods that effectively preserve the RNA of viruses at room temperature for an extensive time period, thereby simplifying and reducing the costs of shipping clinical specimens or isolates to laboratories.

Recently, several new products, such as RNAstable (Biomatrica, San Diego, CA, USA), GenTegra (IntegenX, Pleasanton, CA, USA) and RNAshell (Imagene, Evry Cedex, France) have been developed for the purpose of preserving RNA at room temperature based on the principle of anhydrobiosis (Mathay et al., 2012). However, these systems require RNA to be pre-extracted, which can be impractical if samples are being sent from a field site or a laboratory with limited technical equipment. In contrast, more traditional methods of nucleic acid preservation have involved blotting of samples onto specially designed filter papers such as ‘Guthrie cards’ (Guthrie and Susi, 1963) or ‘Nobuto strips’ (Dusek et al., 2011). These have the advantage of not requiring sample preparation or specialized equipment at the point of sample collection, but they have been primarily used for the collection and analysis of blood (Zhou et al., 2006; Michaud et al., 2007) or serum samples (WHO, 2006), and not respiratory specimens.

The Whatman FTA card (GE Healthcare, Rydalmere, NSW, Australia) is another filter paper system that differs from regular filter paper, as it contains chemicals that can inactivate viruses and stabilize nucleic acids. However, these appear to have been designed specifically for long-term preservation of DNA at room temperature, as the manufacturer recommends that RNA be processed as soon as it reaches the laboratory or be kept frozen (Sigma-Aldrich, 2016). Nevertheless, the Whatman FTA cards have been used in the collection and storage of RNA from cloacal and oropharyngeal swabs for the detection of avian influenza (Abdelwhab et al., 2011; Keeler et al., 2012), and reported to be successful in inactivating viruses and stabilizing RNA for up to 5 months (Abdelwhab et al., 2011).

The RNA*Sound* card (FortiusBio LLC, San Diego, CA, USA) is similar to the Whatman FTA card, but with a simpler RNA extraction/elution process. Virus samples are inactivated once applied to the disks of the RNA*Sound* card and easily eluted by pushing-out the disks and shaking them in RNase-free water. In comparison, special reagents, equipment and techniques are required to isolate RNA from the Whatman FTA cards (Kraus et al., 2011). In this study, we evaluated the efficiency of the RNA*Sound* card for RNA preservation of cell culture-grown influenza viruses. The successful preservation of RNA was assessed by real-time RT-PCR analysis and Sanger sequencing, and was compared to applying the same virus samples or their purified RNA to filter paper, or storing the material in liquid form at different temperatures and durations.

## Materials and Methods

### Viruses

Two influenza virus isolates, a type A(H1N1)pdm09 virus (A/South Australia/17/2013) and a type B (B/Victoria/02/1987-lineage) virus (B/Brisbane/60/2008), were collected as part of the WHO Global Influenza Surveillance and Response System and cultured in Madin-Darby canine kidney (MDCK) cells. These viruses were handled in a PC2 facility to comply with biohazard requirements. Both viruses were diluted in phosphate buffered saline (PBS) to a titre of 32 haemagglutination units (per 25 μl). These were then also used to prepare purified RNA in a 1:1 volume ratio.

### Application of Virus or RNA to RNA*Sound* Card and Other Storage Media

**FIGURE 1:**
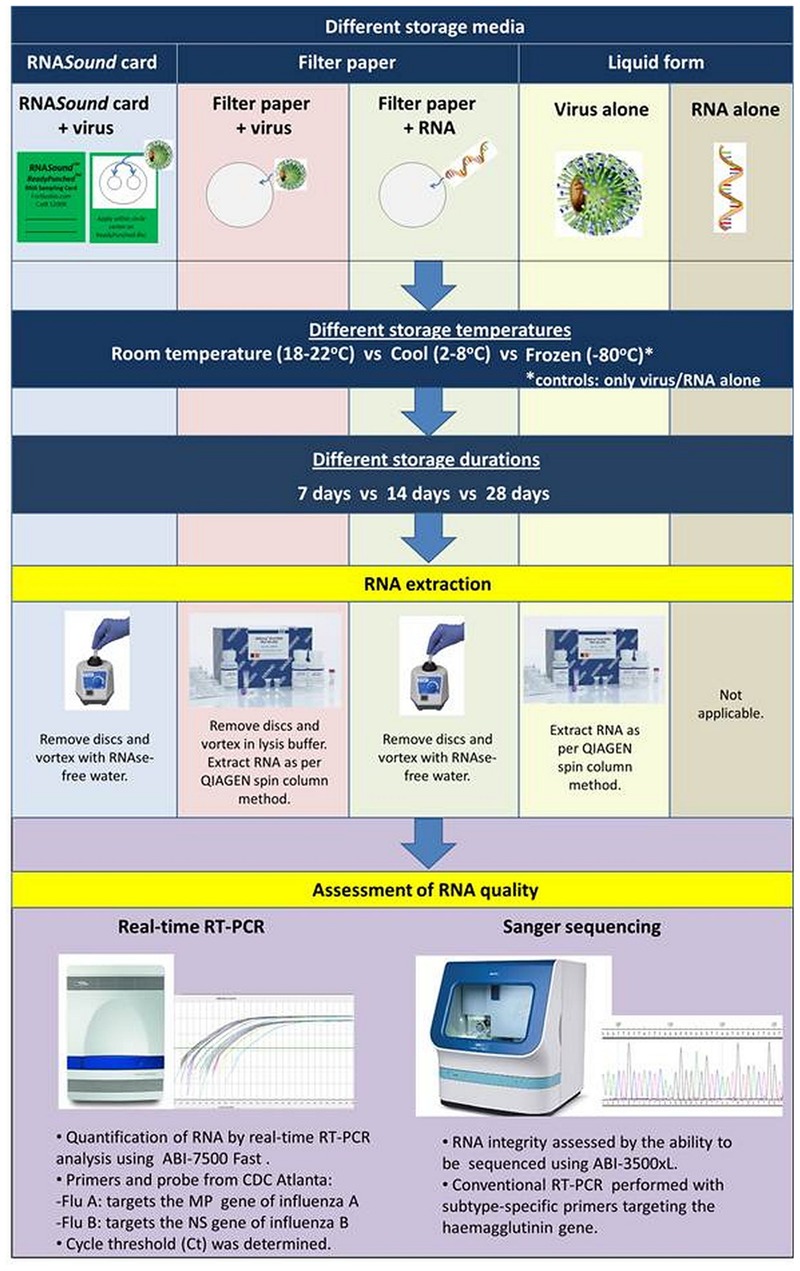
**Study methodology.** Influenza virus or RNA was stored in different media (RNA*Sound* card, filter paper, or liquid form) at different temperatures (cool or room temperature) and durations (7, 14, or 28 days). The quality of RNA recovered from these test conditions were analyzed by real-time RT-PCR and Sanger sequencing.

The different storage conditions used to assess the RNA*Sound* card are summarized in **Figure 1**. Briefly, virus was applied to either the RNA*Sound* card or Whatman Grade No. 1 filter paper (GE Healthcare, Rydalmere, NSW, Australia), or kept in liquid form, and all were stored at either room temperature (18–22°C) or in a fridge (2–8°C; and in the case of the liquid form, also stored at -80°C), for either 7, 14, or 28 days. Purified RNA was also stored under the same conditions following either application to filter paper or being kept in liquid form. Unless otherwise indicated, all RNA purifications/extractions were performed using the QIAamp Viral RNA kit (Qiagen, Chadstone, VIC, Australia). Ten microliters of virus or purified RNA (for filter paper only) was applied onto each of the two disks of the RNA*Sound* card or onto each of two similarly sized disks of filter paper. All disks were left to air-dry for an hour at room temperature, then stored in individual sealed plastic bags containing a desiccant, as per manufacturer’s instructions. For the liquid form, virus (cell culture isolate supernatant diluted in PBS) or RNA (in RNase-free water) was stored in 20 μl aliquots. To recover RNA from virus applied to the RNA*Sound* card, the two push-out disks per sample were placed into a 1.5 ml microtube containing 100 μl pre-heated (75°C) RNAase-free water, shaken for 5 min using a vortex, and then the disks were removed. For RNA recovery of virus applied to the filter paper, the two disks were placed into a 1.5 ml microtube containing AVL buffer + carrier RNA (Qiagen, Chadstone, VIC, Australia), shaken for 5 min using a vortex, and then RNA was extracted as per manufacturer’s protocol, but with a final elution volume of 100 μl using RNAse-free water. RNA recovered from virus stored in liquid form, including the control virus stored at -80°C, was eluted in 100 μl RNAse-free water. For the purified RNA that was applied directly to filter paper, the same method to that used for eluting from the RNA*Sound* card was used, where two disks were placed into a 1.5 ml microtube containing 100 μl pre-heated (75°C) RNAase-free water, shaken for 5 min, and then the disks were removed. Purified RNA stored in liquid form (20 μl), including the -80°C control purified RNA, was made up to a total volume of 100 μl with RNase-free water, to ensure the dilution factor was equivalent to the other methods. With the exception of the -80°C controls, all eluted RNA was used immediately on the same day for real-time RT-PCR analysis and Sanger sequencing, thus eliminating any possible freeze-thaw effects on the RNA.

### Quality Assessment of RNA Recovered from the Various Storage Methods

To assess the quality of RNA, a real-time RT-PCR was performed using the SensiFAST Probe Lo-ROX One-Step kit (Bioline, Alexandria, NSW, Australia) according to the manufacturer’s protocol with 4 μl of RNA used per reaction. Primer/probe sets targeting the matrix (MP) segment of the influenza A virus (FluA) and the non-structural (NS) segment of the influenza B virus (FluB) were kindly provided by the Influenza Division at CDC, Atlanta, USA (CDC, 2012a,b). Thermocycling parameters were 45°C for 10 min, then 95°for 2 min, followed by 40 cycles of 95°C for 5 s and 60°C for 30 s. Each sample was run in triplicate and analyzed using the 7500 Real Time PCR System (Applied Biosystems, Foster City, CA, USA). The mean cycle threshold (*C*_t_) value for each sample held under various conditions was compared with the mean *C*_t_ value for the respective control virus or purified RNA stored at -80°C (considered the gold standard for storage of viruses and nucleic acids). Analysis of variance and *post hoc* Tukey test were used to compare mean *C*_t_ values and generate 95% confidence intervals for the difference in mean *C*_t_ (Δ*C*_t_) to the -80°C control. All analyses were performed using Stata14 (StataCorp, 2015).

In addition to real-time RT-PCR analysis, the preservation of RNA was also assessed by Sanger sequencing. Conventional RT-PCR was performed using the MyTaq One-Step RT-PCR kit (Bioline, Alexandria, NSW, Australia) using in-house subtype-specific HA primers according to the manufacturer’s protocol. Thermocycling conditions were 45°C for 40 min, 95°C for 1 min, followed by 40 cycles of 95°C for 10 s, 60°C for 10s and 72°C for 1 min, with a final hold at 72°C for 2 min. RT-PCR products were visualized on an E-Gel (Invitrogen, Carlsbad, CA, USA) and purified by ExoSAP-IT (Affymetrix, Cleveland, OH, USA). A sequencing reaction was then conducted using Big Dye Terminator Reaction Mix (Applied Biosystems, Carlsbad, CA, USA) and purified with Big Dye Xterminator (Applied Biosystems, Carlsbad, CA, USA) before being run on the ABI 3500xL sequencer. Sequence data was analyzed using the SeqMan (DNASTAR Lasergene9, Madison, WI, USA) software.

## Results

### Real-Time RT-PCR Amplification

**FIGURE 2:**
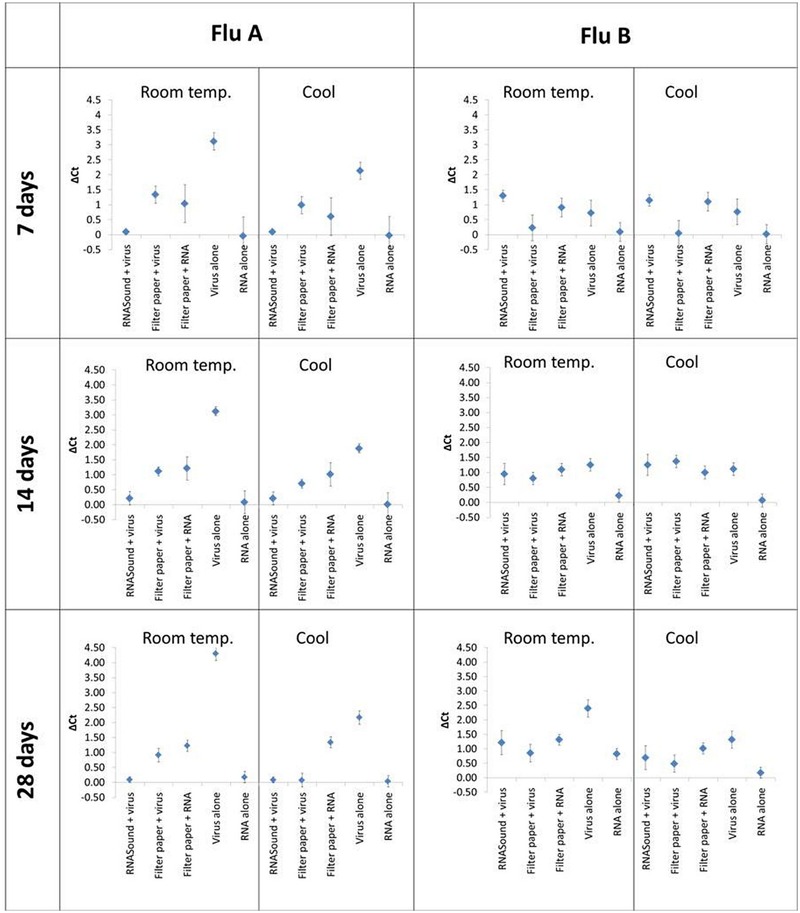
**Difference in mean *C*_t_ (Δ*C*_t_) values of different sample test groups compared to control viruses/RNA stored at 80°C.** Sample test groups consist of influenza A/B viruses/RNA applied to different storage media, stored at cool/room temperature for 7,14, or 28 days. Error bars represent 95% confidence interval.

Data presented in **Figure 2** summarizes the difference in mean *C*_t_ values (Δ*C*_t_) between the *C*_t_ values of RNA eluted from the RNA*Sound* card (and other storage media) compared with the ‘gold standard’ of virus stored at -80°C for the equivalent amount of time. The preservation of influenza A virus on the RNA*Sound* card for 7, 14, or 28 days at room temperature was highly effective with *C*_t_ values within <0.5 cycles of virus stored at -80°C and extracted using standard techniques (**Figure 2**). No additional benefit was observed if the RNA*Sound* cards were kept cool rather than at room temperature. The preservation of influenza A virus on Whatman Grade No. 1 filter paper (followed by standard RNA extraction) yielded *C*_t_ values that were ≈1 *C*_t_ value higher than the virus stored at -80°C across all time points and temperatures, indicating only a minor loss in RNA quantity (**Figure 2**). The addition of purified RNA to Whatman Grade No. 1 filter paper also showed a minor loss in quantity compared to the purified RNA control stored at -80°C. As expected, virus stored in liquid form showed the worst deterioration at room temperature with increases of ≈3 *C*_t_ values after 7 and 14 days, and ≈4 *C*_t_ values after 28 days, compared to virus stored at -80°C (**Figure 2**). However, purified RNA in liquid form (RNase-free water) appeared very stable at both cool and room temperatures for up to 28 days, yielding highly similar *C*_t_ values (within <0.5 cycles) to purified RNA stored at -80°C for the equivalent amount of time (**Figure 2**).

There was a minor loss of RNA quantity (≈1 *C*_t_ value) following preservation of the influenza B virus on the RNA*Sound* card for 7, 14, or 28 days at cool or room temperature, compared to virus stored at -80°C. Interestingly for short-term (7 days) storage, virus on filter paper appeared to perform better than the RNA*Sound* card, although there was no significant difference between the two at 14 or 28 days. Purified RNA on filter paper behaved similarly to virus on the RNA*Sound* card with a minor loss in quantity. Influenza B virus stored in liquid form appeared more stable than influenza A virus, with increases of ≈1 *C*_t_ value at 7 and 14 days, and ≈2 *C*_t_ values at 28 days, compared to virus stored at -80°C. Purified RNA (in RNase-free water) was again very stable at cool or room temperature, with only a slight increase of <1 *C*_t_ value when stored at room temperature for 28 days, compared to purified RNA stored at -80°C for the equivalent amount of time.

### Sanger Sequencing Analysis

**FIGURE 3:**
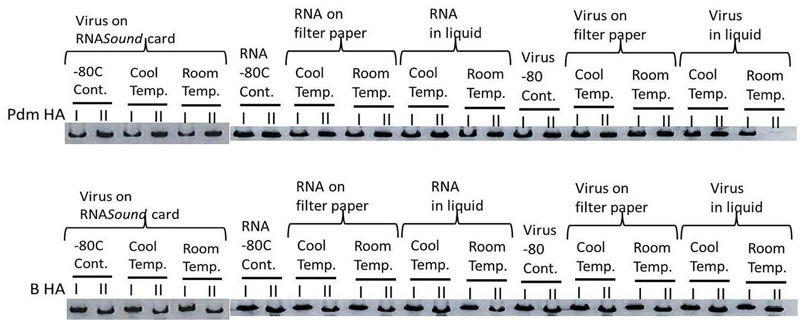
**Representative E-Gel image of RT-PCR products (from day 28) amplified from RNA recovered from the various storage method, as compared to the -80 control for RNASound card, filter paper and liquid**.

Conventional RT-PCR of the HA gene was conducted on RNA from both the influenza A and B viruses preserved under the various conditions outlined above. Amplicons of a similar intensity to that obtained from the -80°C controls were successfully generated for all samples, as visualized following gel electrophoresis, except for the influenza A virus stored in liquid form at room temperature for 28 days, where the amplicon was faint on the E-Gel (**Figure 3**). Subsequent sequence analyses of the RT-PCR products showed that the electropherogram and sequence quality were of a high standard for all samples except for the influenza A virus stored in liquid form at room temperature for 28 days (data not shown).

## Discussion

Although substantial influenza surveillance occurs within well-resourced healthcare settings, there are many situations, such as respiratory outbreaks or testing in remote locations, where immediate access to laboratories can be difficult. In such cases, simple and effective methods for the collection and shipment of samples for rapid diagnosis are important. If virus samples are unable to be shipped under cool (2–8°C) or frozen (-80°C) conditions promptly, then the virus and its nucleic acid can rapidly degrade, potentially impacting on the capability of laboratories to achieve a diagnosis.

Many commercially available products have been developed for the purpose of preserving RNA at room temperature, which allows for easier storage and shipping. We chose to test the RNA*Sound* card in this study due to its ease-of-use in preserving RNA at room temperature, its ability to inactivate viruses when applied to the card, and the simple RNA extraction process. Although this is the first reported use of the RNA*Sound* card for preserving influenza virus RNA, there has been an established protocol of its use for West Nile Virus RNA preservation as part of a dead bird surveillance program (CDPH, 2016), in which the RNA*Sound* cards containing samples were placed in an envelope and shipped via regular mail. A more recent study that evaluated two different dry plasma transport systems for preserving HIV-1 RNA at ambient temperature reported higher effectiveness of the RNA*Sound* card compared to ViveST tubes (Levine et al., 2016). Aside from the shipping of clinical specimens for diagnostic purposes, the RNA*Sound* card may also be used to rapidly share inactivated reference viruses/RNA between laboratories to assist in the establishment of assays at the start of outbreaks, such as during the start of the 2009 influenza pandemic.

In this study, we evaluated the capability of the RNA*Sound* card to preserve RNA of MDCK-cultured influenza viruses and we found that it performed well with little or no degradation of the RNA. Our results did not indicate a clear superiority between the RNA*Sound* card and Whatman Grade No. 1 filter paper in terms of RNA preservation, but these were definitely better than storing viruses in liquid form. Among the limitations of this study was that only small regions of the RNA were amplified by the RT-PCR assays and certain sections or segments of the influenza virus genome may not be preserved as well as the regions investigated here. However, further assessment may be necessary to test the effectiveness of the RNA*Sound* card for preservation of viral RNA in original specimens, as there is potential for RNases or inhibitors to alter effectiveness when testing certain specimen types. In addition, further evaluation of RNA preservation at higher temperatures that those tested here (e.g., 37°C), which could occur during sample shipment, and the effect of varying virus concentrations to determine the quantity of RNA that can be eluted from the card, may be worthwhile. Furthermore, while the inactivation of viruses upon application to the RNA*Sound* card can, in certain circumstances, be advantageous as it reduces the biohazardous nature of infectious viruses, it has the disadvantage that the specimen is no longer viable for culture in the laboratory. Although RNA alone is sufficient for diagnostic tests, a more detailed antigenic characterization of influenza viruses requires an egg- or cell-cultured isolate. Another concern is that the inactivation may be dependent on the amount of virus added to the card. An interesting side observation from this study was that RNA eluted in RNase-free water was extremely stable at room temperature and therefore may indicate that purified RNA in RNase-free water does not need to be shipped on dry ice.

Overall, we observed that the RNA*Sound* card was effective in preserving influenza RNA when cell culture isolates of influenza A and B viruses were applied to the card, although the effectiveness differed slightly depending on the virus used. Future studies that test additional influenza A and B viruses may confirm, or otherwise, the differences between viruses of the two types for the RNA*Sound* card vs. filter paper on day 7. Despite also observing a similar performance with virus applied to filter paper, we conclude that the main benefit in using the RNA*Sound* card lies in its ability to inactivate viruses (due to the presence of lysis buffer) and its simple RNA recovery method (without having to undergo the cost or time of conventional RNA extraction processes). As the RNA*Sound* card can inactivate viruses and preserve RNA at room temperature for up to 28 days, this makes it feasible to send RNA using regular mail. There will be no need for expensive shipping conditions requiring biohazard containers and dry ice. Moreover, the quick and simple RNA recovery from the RNA*Sound* card allows recipient labs to extract the RNA for testing without the need for special reagents or equipment.

## Author Contributions

AH and HL conceived the study, conducted the experiments, then drafted, revised and approved the manuscript.

## Conflict of Interest Statement

The authors declare that the research was conducted in the absence of any commercial or financial relationships that could be construed as a potential conflict of interest.

FortiusBio LLC, San Diego, CA, USA provided the RNA*Sound* RNA Sampling cards free-of-charge for use in the evaluation. FortiusBio did not provide any funding for the study, and had no input into the experimental design, analysis of results or the preparation of the manuscript.
